# Substitution of manure for mineral P fertilizers increases P availability by enhancing microbial potential for organic P mineralization in greenhouse soil

**DOI:** 10.3389/fbioe.2022.1078626

**Published:** 2022-12-06

**Authors:** Ruibo Sun, Junfang Niu, Bingbing Luo, Xiaogai Wang, Wenyan Li, Wenjie Zhang, Fenghua Wang, Chaochun Zhang, Xinxin Ye

**Affiliations:** ^1^ Anhui Province Key Lab of Farmland Ecological Conservation and Pollution Prevention, Engineering and Technology Research Center of Intelligent Manufacture and Efficient Utilization of Green Phosphorus Fertilizer of Anhui Province, Research Centre of Phosphorous Efficient Utilization and Water Environment Protection Along the Yangtze River Economic Belt, College of Resources and Environment, Anhui Agricultural University, Hefei, China; ^2^ Key Laboratory of JiangHuai Arable Land Resources Protection and Eco-restoration, Ministry of Natural Resources, Hefei, China; ^3^ Key Laboratory of Agricultural Water Resources, Hebei Key Laboratory of Soil Ecology, Center for Agricultural Resources Research, Institute of Genetics and Developmental Biology, Chinese Academy of Sciences, Shijiazhuang, China; ^4^ School of Life Science and Engineering, Handan University, Handan, China; ^5^ Xiong’an Institute of Innovation, Chinese Academy of Sciences, Shijiazhuang, China; ^6^ Hebei Key Laboratory of Environmental Change and Ecological Construction, Hebei Experimental Teaching Demonstrating Center of Geographical Science, School of Geographical Sciences, Hebei Normal University, Shijiazhuang, China; ^7^ College of Resources and Environmental Sciences, National Academy of Agriculture Green Development, Key Laboratory of Plant-Soil Interactions, Ministry of Education, China Agricultural University, Beijing, China

**Keywords:** P fertilizer, manure, bacterial community, organic P mineralization, greenhouse soil, phosphate solubilizing microbes

## Abstract

The shortage of phosphorus (P) as a resource represents a major challenge for the sustainable development of agriculture. Manure has a high P content and is a potential substitute for mineral P fertilizers. However, little is known about the effects on soil P availability and soil microbial P transformation of substituting manure for mineral P fertilizers. In this study, variations in soil P availability and bacterial P mobilization were evaluated under treatment with manure as compared to mineral P fertilizers. In the greenhouse fruit and vegetable production system that provided the setting for the study, substitution of manure for mineral P (PoR treatment) resulted in a similar level of soil total P and a similar fruit and vegetable yield as compared to traditional fertilization, but a significantly increased level of soil available P. In addition, PoR treatment enhanced bacterial organic P mineralization potential and decreased inorganic P dissolution potential. These results demonstrate that manure application increases the availability of soil P primarily by enhancing soil microbial Po mineralization, indicating the potential feasibility of applying manure instead of mineral P fertilizers in greenhouse farming.

## Introduction

Phosphorus (P) is a fundamentally important element in agricultural production. Around 80% of phosphate rock use occurs in agricultural systems in fertilizers, and total global P consumption is expected to continue increasing (Van Vuuren et al., 2010). Thus, a shortage of P fertilizers may greatly restrict the development of agriculture in the near future, as phosphate rock is a non-renewable resource. Effective management of phosphorus resources and reduction of the dependence on mineral P fertilizers is of great importance for the sustainable development of agriculture, as well as for society as a whole (Withers et al., 2014; Fu et al., 2021; Rong et al., 2021; Xu et al., 2021; Zheng et al., 2022). Many approaches are considered to have potential to reduce mineral P input, one of which is recycling of the P content of animal excreta (Van Vuuren et al., 2010; Withers et al., 2014).

Global animal stocks are extremely large. Livestock production greatly impacts global nutrient cycles, and improved integration of animal manure into crop production can effectively reduce nutrient flows (Bouwman et al., 2013). Manure has a high P content and contains a variety of forms of P, including both organic P (Po) and inorganic P (Pi), and nearly 70% of the total P in manure is labile (Shen et al., 2011); thus, manure represents a good substitute for mineral P fertilizers. In addition, manure also contains various organic substances that could reduce P adsorption to soil particles, thereby increasing soil P availability. Finally, manure can also alter soil P availability by modifying the biochemical conditions of the soil, such as its pH (Shen et al., 2011).

Soil microbes play a vital role in regulating soil P dynamics and bioavailability (Chen H. M. et al., 2019; Lai et al., 2022), especially in organic P mineralization (Weihrauch and Opp, 2018), as well as in influencing P uptake by plants (Zhang et al., 2021). Not only is it possible that manure application may alter soil P pools, but it may also shape the constitution of the soil microbial community (Sun et al., 2020). Our previous study has revealed that the high levels of easily degradable carbon found in manure significantly enhance microbial Po mineralization through enrichment of Po-mineralizing microbial taxa (Chen et al., 2020), suggesting that support of soil microbial function in phosphorus cycling is a key mechanism in enabling manure application to modify soil P availability.

Greenhouse farming is an important aspect of agricultural production. Through recent advancements in greenhouse technology, greenhouse farming has become increasingly productive and is considered to be a promising approach to ensuring that demand for food is satisfied in future (Aznar-Sánchez et al., 2020). However, as a resource-intensive production system, greenhouse farming requires heavy fertilizer input; this makes it highly challenging in terms of mineral resources, especially non-renewable ones, such as phosphate rocks. As mentioned above, manure has a high P content, and it has great potential to function as an alternative to mineral P fertilizers. However, the composition of P in manure in terms of form differs greatly from that of P in mineral P fertilizers. Therefore, there is a need to evaluate the impact of manure application on soil P availability and soil microbial P transformation; doing so can provide valuable information on the possibility of replacing mineral P fertilizers with manure in greenhouse production.

## Methods

### Experimental design

A field experiment was conducted to investigate the impact of replacement of mineral P with manure on soil P availability and microbial phosphorus transformation. Two treatments were implemented as part of the experiment: one (the control) consisting of conventional fertilization with mineral nitrogen (N, 90 kg N·ha^−1^·year^−1^), P (90 kg P_2_O_5_·ha^−1^·year^−1^), and potassium (K, 90 kg K_2_O·ha^−1^·year^−1^) fertilizers and cattle manure (84 t ha^−1^·year^−1^), and the other (PoR treatment) consisting of Po fertilization with manure (134.4 t ha^−1^·year^−1^) in place of the mineral P fertilizer. The total input of P under the PoR treatment was same as under the control. Implementation of the experiment began in 2017 in a solar greenhouse located in Raoyang County, Hebei Province, China (38°15′N, 115°44′E). The soil was silt loam. Each treatment was replicated across three plots, and the plant system was a tomato and muskmelon rotation.

### Soil sampling and measurement of soil properties

On 10 June 2020, surface soil (0–20 cm) was collected from each plot in accordance with procedures described in a previous study (Sun et al., 2015). The soil samples were sieved though a 2 mm sifter to mix thoroughly and remove impurities, such as plant roots and stones.

Soil pH, total carbon (TC), total nitrogen (TN), soil organic matter (SOM), ammonia (NH_4_^+^–N), nitrate (NO_3_^−^–N), available P (AP), and total P (TP) were measured as described in a previous study (Sun et al., 2022).

### DNA extraction and high-throughput sequencing

Soil total DNA was extracted from 0.5 g fresh soil using a FastDNA Spin Kit for Soil (MP Biomedicals, Santa Ana, CA, United States).

The soil bacterial community was characterized using high-throughput sequencing as described in a previous study (Sun et al., 2018). In brief, primer sets 515F/806 R targeting the V4 region of the bacterial 16S rRNA gene were used for polymerase chain reaction (PCR) analysis. PCRs were performed in a 50-µL mixture containing 25 µL PCR premix (TaKaRa Ex TaqR), 1 µL forward primer (10 µM), 1 µL reverse primer (10 µM), 1 µL DNA template (20 ng), and 22 µL PCR-grade water under the following conditions: initial denaturation at 94°C for 10 min; 30 cycles in a series of denaturation at 94°C for 1 min, annealing at 50°C for 1 min, and extension at 72°C for 1 min; and a final extension at 72°C for 10 min. After quality checks and purification, the PCR products were sequenced using an Illumina HiSeq 2,500 system.

### Determination of microbial P transformation profiles

Soil bacterial functional profiles with respect to P cycling were predicted using PICRUSt2 (Phylogenetic Investigation of Communities by Reconstruction of Unobserved States) (Douglas et al., 2020). Genes involved in P transformation were extracted to reveal the potential capacity for microbial P transformation under each of the treatments applied.

Soil alkaline phosphatase activity was measured using a Soil Alkaline Phosphatase (S-AKP/ALP) Activity Assay Kit (Beijing Solarbio Science & Technology Co., Ltd. China), and expressed in the form of nM *p*-PNP (*p*-nitrophenyl phosphate) h^−1^g^−1^ dry soil. The abundance of the *phoD* gene, which encodes for alkaline phosphatase, was determined using real-time fluorescent quantitative PCR (qPCR), which was conducted using primer sets phoD-F733/phoD-R1083 (5′-TGGGAYGATCAYGARGT-3′/5′-CTGSGCSAKSACRTTCCA-3′) under the following amplification conditions: 10 min at 95°C, followed by 40 cycles of denaturation at 95°C for 15 s and annealing at 55°C for 1 min. After amplification, melting curve analysis and gel electrophoresis were performed to measure the specificity of the reaction (Chen X. et al., 2019).

The microbial dissolving potential for calcium phosphate was determined for each treatment using an incubation method, in accordance with procedures described in our previous study (Sun et al., 2022). Briefly, 1 g soil was added to 90 ml sterilized water and mixed thoroughly. Next, 1 ml of the mixture was added to 50 ml sterile PVK liquid medium and incubated at 28°C (180 rpm) for 5 days. A blank control with 1 ml sterilized water was included. Dissolved P in the culture was measured at 0,12, 24, 48, 72, 96, and 120 h.

### Bioinformatic analysis of the high-throughput sequencing data

VSEARCH (version 2.21.1) (Rognes et al., 2016) was used for bioinformatic analysis of the high-throughput sequencing data following the protocols described in previous work (Liang et al., 2020; Sun et al., 2022). The paired-end reads were first merged, and low-quality and chimeric reads were then removed. Subsequently, the clean reads were further denoised and zOTUs (zero-radius operational taxonomic units) were generated using the UNOISE algorithm (version 3). The taxonomic details of each zOTU were determined using the RDP Classifier tool based on the SILVA rRNA database (version 138). After removal of zOTUs not annotated as bacteria, the zOTU tables were subsampled to extract 48,000 reads per sample for statistical analysis.

### Statistical analysis

Statistical analysis was conducted and figures generated using R (version 4.0.2) as described in our previous study (Sun et al., 2022). The Kruskal–Wallis rank sum test was used to test for significant differences between treatments for each variable. Principal coordinate analysis (PCoA) was performed based on Bray–Curtis distance using the “vegan” library.

## Results

### Effects of PoR treatment on soil properties and crop yield

PoR treatment significantly lowed soil pH, but had no significant impact on soil contents in terms of total carbon (TC), total nitrogen (TN), soil organic matter (SOM), ammonia nitrogen (NH_4_^+^–N), or nitrate nitrogen (NO_3_^−^–N) (Table 1). Levels of soil total P (TP) were similar under the two treatments (Table 1), while PoR treatment significantly increased the level of soil available P (AP), which was 35.51% higher in samples having undergone PoR treatment compared to the control (Table 1).

**TABLE 1:** Soil properties and crop yield following different treatments.

Treatment	pH	TC (%)	TN (%)	SOM (%)	NH_4_^+^–N (mg·kg^−1^)	NO_3_^−^–N (mg·kg^−1^)	AP (mg·kg^−1^)	TP (g·kg^−1^)	Tomato yield (t·ha^−1^)	Muskmelon yield (t·ha^−1^)
Control	7.82 (0.15)a	2.50 (0.06)b	0.18 (0.02)a	1.54 (0.08)a	1.41 (0.56)a	17.39 (0.19)a	179.79 (16.57)b	2.17 (0.08)a	132.80 (1.20)a	85.70 (6.90)a
PoR	7.62 (0.04)b	2.51 (0.08)a	0.20 (0.01)a	1.57 (0.04)a	1.30 (0.45)a	17.74 (1.15)a	243.63 (13.02)a	2.22 (0.10)a	126.50 (8.00)a	88.30 (1.40)a

Data was averages with standard deviations in (brackets), *n* = 3.

TC, total carbon; TN, total nitrogen; SOM, soil organic matter; NH_4_^+^–N, ammonia nitrogen; NO_3_^−^–N, nitrate nitrogen; AP, available P; TP, total P.

Different lowercase letters beside data points in the same column indicate a significant difference between treatments according to the Kruskal–Wallis rank sum test (*p* < 0.05).

Tomato and muskmelon yields did not differ significantly between the control and PoR plots (Table 1), indicating that PoR treatment has a similar productivity to that of conventional fertilization.

### Variation in microbial communities under different treatments

**FIGURE 1:**
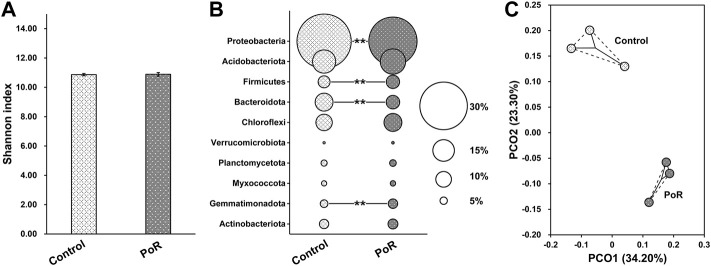
Shannon diversity **(A)** and taxonomic composition **(B)** of the bacterial community under different treatments; PCoA plot **(C)** showing variation in the bacterial community under different treatments. ** indicates a significant difference between PoR and control (Kruskal–Wallis rank sum test, *p* < 0.01).

The Shannon index was calculated as a measure of variation in bacterial diversity. The results showed that there was no significant difference in bacterial α-diversity between samples having undergone control and PoR treatment (Figure 1A), indicating that PoR treatment had little impact on bacterial α-diversity. However, soil bacterial community composition was impacted by PoR treatment. Proteobacteria was the most dominant phylum in the control soil, but it featured in a significantly lower proportion in the PoR soil. Bacteroidota was also diluted under PoR treatment, while Firmicutes and Gemmatimonadota were enriched (Figure 1B). This variation in bacterial community structure is further illustrated by the corresponding 2D PCoA plot (Figure 1C), which shows the clear separation in bacterial community between control and PoR samples. ANOSIM (Analysis of Similarities) also confirmed that there was a significant difference between control and PoR samples in terms of bacterial community (*p* < 0.05).

### Effects of PoR treatment on microbial P-mobilization profiles

**FIGURE 2:**
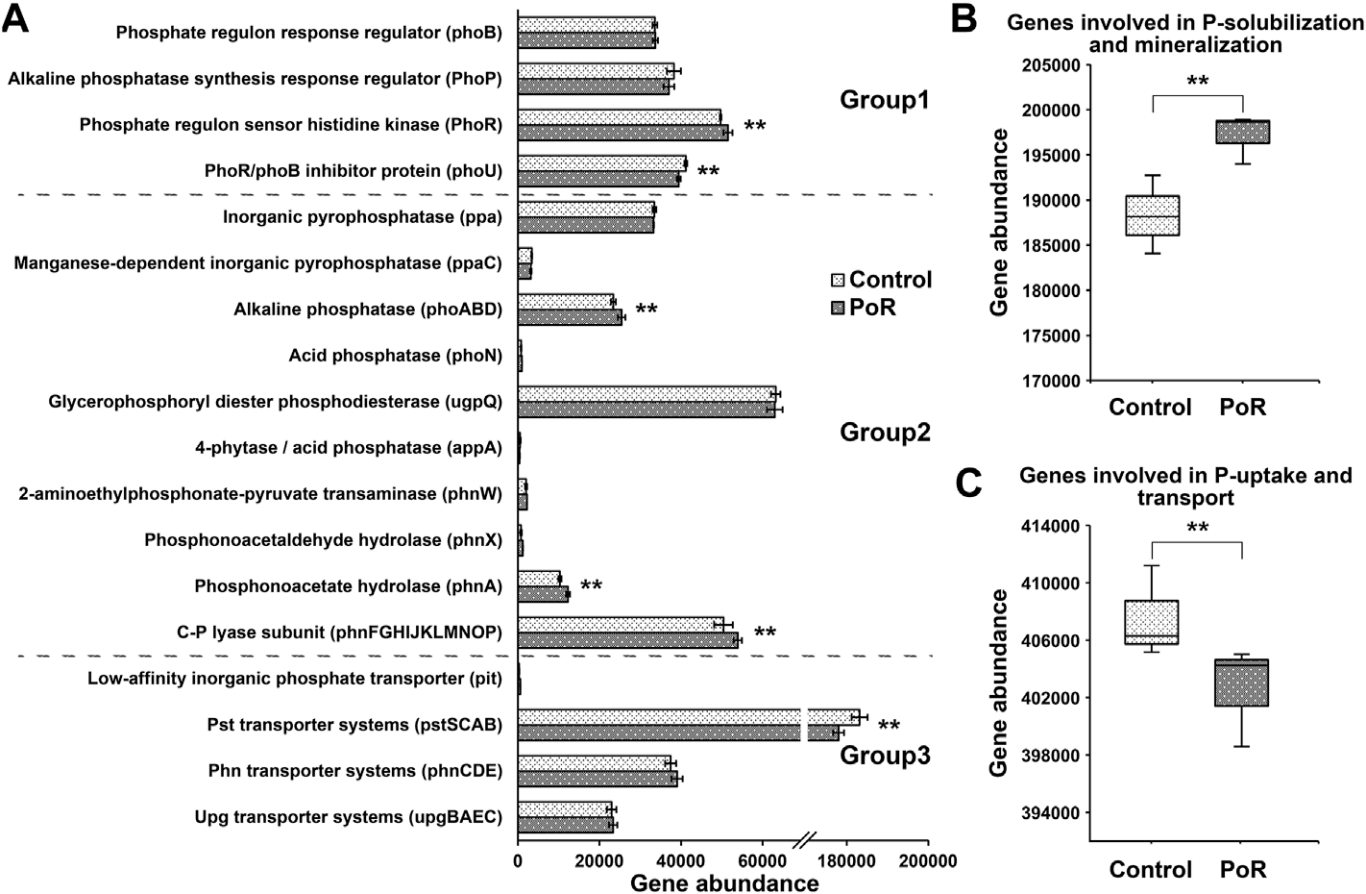
The abundance of genes involved in P transformation in soils having undergone different treatments **(A)**. Group 1: genes coding for regulation of the P-starvation response; Group 2: genes coding for inorganic P-solubilization and organic P-mineralization; Group 3: genes coding for P-uptake and transport. Total abundance of genes involved in P-solubilization and mineralization **(B)** and P-uptake and transport **(C)**. ** indicates a significant difference between PoR and control (Kruskal–Wallis rank sum test, *p* < 0.01).

Through functional prediction (using PICRUSt2), 38 genes involved in P transformation were detected (Park et al., 2022). PoR treatment significantly decreased the abundance of the *phoU* and *pst* genes, while it significantly increased the abundance of the *phoR* and *phnA* genes and genes coding for alkaline phosphatase and C-P lyase (Figure 2A). Considering genes in terms of functional groups, those involved in Pi solubilization and Po mineralization were significantly increased under PoR treatment (Figure 2B). In contrast, the abundance of genes involved in P uptake and transport was significantly decreased under PoR treatment (Figure 2C).

**FIGURE 3:**
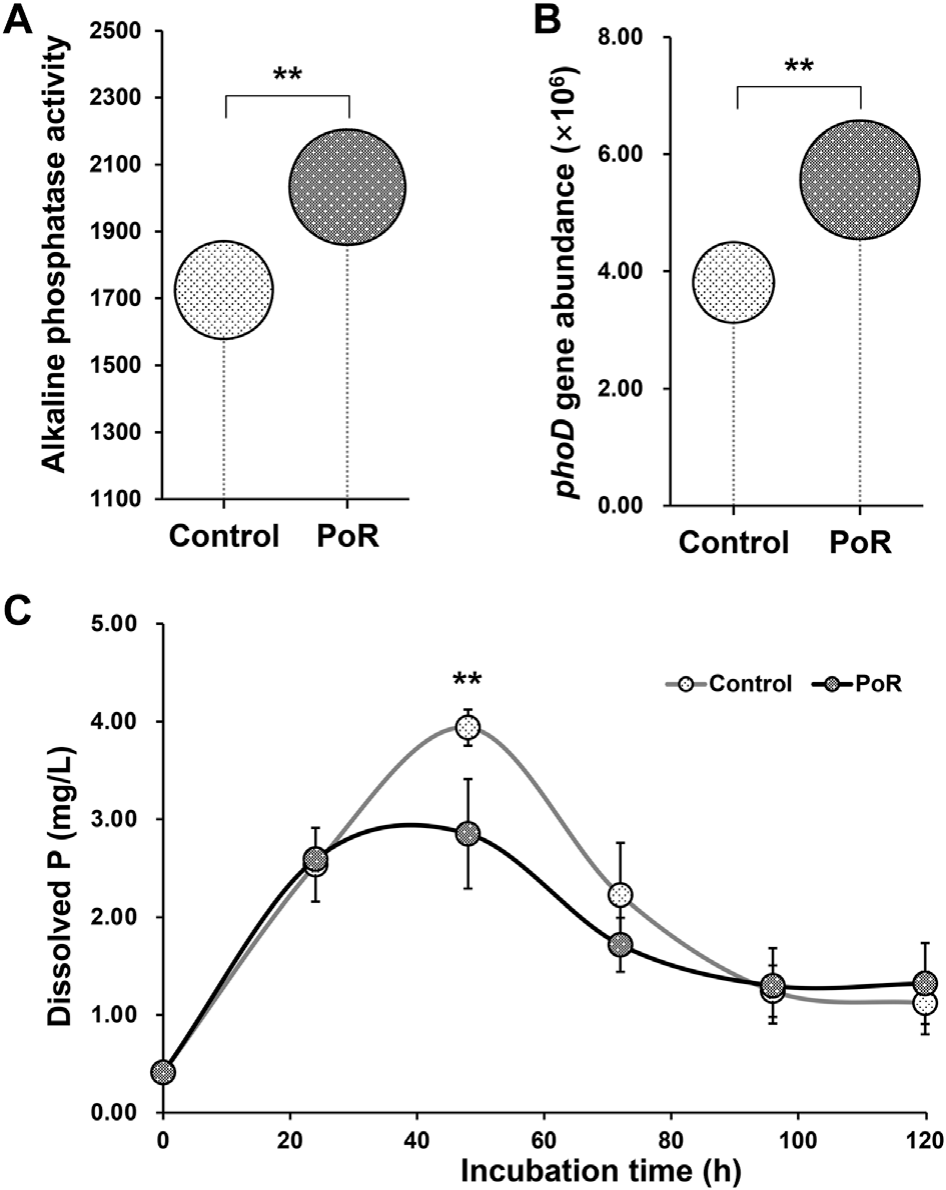
Alkaline phosphatase activity (nM *p*-NPP h^−1^ g^−1^ dry soil) **(A)** and the abundance of the *phoD* gene (number of copies g^−1^ dry soil) **(B)** in soil having undergone PoR and control treatments; dynamics of inorganic P-solubilization by microbes under control and PoR treatments **(C)**. ** indicates a significant difference between PoR and control (Kruskal–Wallis rank sum test, *p* < 0.01).

Soil alkaline phosphatase activity was significantly higher under PoR treatment than under control treatment (Figure 3A). A similar pattern was observed in the abundance of the *phoD* gene, which was significantly increased by PoR treatment (Figure 3B).

Taking calcium phosphate as the sole P resource, the incubation experiment indicated that, over the entire incubation period, the levels of dissolved P in the culture liquid followed an approximate bell curve, peaking at 48 h (Figure 3C). At the 48 h time point, the dissolved P content of the culture with PoR-sourced microbes was significantly lower than that of the culture with control-sourced microbes, showing that the potential P solubilization of the microbial community was significantly lower under PoR treatment than under the control treatment.

## Discussion

As a typical form of intensive production system, greenhouse agriculture depends on heavy resource input; thus, a shortage of resources would represent a major challenge to its productivity. In the present study, we have demonstrated that PoR treatment significantly increases soil P availability without exerting an adverse effect on other nutrient levels or on crop yield, indicating that the substitution of manure for mineral P fertilizers is feasible in greenhouse production.

The increase in P availability in soil that has undergone PoR treatment may be partly associated with the significant decrease in soil pH (Table 1). Manure contains a large amount of organic matter. However, in the present study, soil TC and SOM did not significantly differ between the PoR and control treatments, indicating strong degradation of the organic matter contained in the manure. Various organic acids would be released during the degradation of this organic matter, thereby decreasing soil pH and consequently enhancing the dilution of inorganic phosphorus compound. An existing study has also found that lower pH enhances the dilution of Ca phosphates in alkaline soil (Devau et al., 2009). In addition to impacting soil pH, organic acids also act as “chelates” for phosphates (Kalayu, 2019), as they contain very large amounts of negative charges, carboxyl, and hydroxyl groups, which decrease the precipitation of Al, Fe, and Ca phosphates by competing with Pi for adsorption sites (Shen et al., 2011).

As the primary driver of P transformation in soil, the soil bacterial community underwent a major shift as a result of PoR treatment. The results of high-throughput sequencing showed that PoR treatment reshaped the soil bacterial community into one with a high potential for P mobilization. This was further confirmed by the greater abundance of *phoD* genes in samples that had undergone PoR treatment; this is the most common alkaline phosphatase gene and is widely used as a molecular marker for the detection of alkaline phosphatase-producing bacteria in soil (Chen H. M. et al., 2019). This finding was coincident with the results of alkaline phosphatase activity measurement, which showed that alkaline phosphatase activity was 17.83% higher under PoR treatment than under the control treatment (Figure 3B). Alkaline phosphatase plays a crucial role in releasing Pi from Po in alkaline soil. Studies have found that bacteria are the primary producers of alkaline phosphatase in soil (Chen X. et al., 2019). The results of this study showed that application of manure enhances the mineralization of Po. However, PoR treatment also decreases the bacterial Pi solubilization potential (Figure 3C), indicating that the high levels of AP in PoR soil are largely the result of Po mineralization, while bacterial Pi solubilization may make little contribution to the increase in P bioavailability.

The impact of manure application on bacterial community structure and P mobilization may be largely associated with the resultant changes in the soil components functioning as resources for the microbial community. The type, quantity, and availability of resources are key regulators of the soil microbial community, as microbial taxa have different preferences in terms of resources (Estrela et al., 2021). A large amount of Po input may enhance the competitiveness of the bacterial taxa involved in Po mineralization, resulting in a bacterial community that harbors a high proportion of Po-mineralizing taxa. As a result, the secretion of alkaline phosphatase is enhanced. In addition, application of manure also leads to variation in the soil bacterial community by introducing a large amount of organic carbon, which is also an important driver of microbial community interactions (Bergk Pinto et al., 2019).

In summary, this work has revealed that substitution of manure for mineral P fertilizer significantly increases P availability in greenhouse soil, which can largely be attributed to the enrichment of Po-mineralizing microbes. The results also indicate that manure is a feasible substitute for mineral P fertilizers in greenhouse farming. However, the high AP content resulting from manure application may increase the risk of P leaching; reducing the amount of manure applied may be a possible way to mitigate this problem, but identification of the appropriate amount of manure to use in substitution is an important issue requiring further study.

## Data Availability

The raw data supporting the conclusions of this article will be made available by the authors, without undue reservation.
